# Cyclic restricted feeding enhances lipid storage in 3 T3-L1 adipocytes

**DOI:** 10.1186/1476-511X-12-76

**Published:** 2013-05-24

**Authors:** Takeshi Hashimoto, Yuriko Endo

**Affiliations:** 1 Faculty of Sport & Health Science, Ritsumeikan University, 1-1-1 Nojihigashi, Kusatsu, Shiga 525-8577, Japan

**Keywords:** Abdominal obesity, Breakfast skipping, Circadian rhythm, Eating behaviors, Lipogenesis

## Abstract

**Background:**

People who skip breakfast have more visceral fat than those who eat breakfast; however, the mechanism underlying this difference is unclear. In this study, we examined 3 T3-L1 adipocytes and assessed 1) whether restricted feeding (i.e., “breakfast skipping”) alters the cyclic expression of brain and muscle aryl hydrocarbon receptor nuclear translocator (ARNT)-like protein 1 (BMAL1) and lipogenic proteins and 2) whether repeated exposure to growth media at the time-points with enhanced lipogenic regulatory signals increases *de novo* lipogenesis and lipid storage.

**Methods:**

Differentiated adipocytes were divided into two groups: a control group and a restricted feeding group, for which incubation with growth medium from ZT 9 to ZT 12 was withheld.

**Results:**

A bout of restricted feeding disrupted the cyclic expression of BMAL1 protein and increased the expression of lipogenic proteins, such as fatty acid synthase and peroxisome proliferator-activated receptor gamma in adipocytes. Furthermore, the repeated exposure to growth media at the time-points with enhanced lipogenic regulatory signals increased *de novo* lipogenesis and lipid storage.

**Conclusion:**

These findings suggest that direct disruption of intracellular molecular clock systems by breakfast skipping and the concurrent changes in the daily cycle of lipogenic proteins in adipocytes, as a consequence of repeated nutrition at the time-points with enhanced lipogenic regulatory signals, would result in increased lipogenesis and lipid storage. These alterations are important molecular mechanisms underlying augmented adiposity induced by breakfast skipping.

## Background

Previously, Alexander et al. reported that breakfast skipping was related to increased visceral fat independent of age, gender, total fat, total lean tissue, and total energy intake in overweight Latino youth [1]. Similar cross-sectional studies have also demonstrated that breakfast skipping is associated with obesity in Hong Kong children, US adults, and Taiwanese adults [2-4]. While remarkably little is currently known about the molecular mechanisms underlying the augmentation of adiposity due to breakfast skipping, a recent study by Yoshida et al. demonstrated that early nocturnal meal skipping in mice disturbed the peripheral clock and increased *de novo* lipid synthesis in liver and fat [5].

It has been reported that peripheral tissues, including adipose tissue, possess autonomous molecular machinery. These tissues respond to a variety of physiological stimuli other than the central clock systems governed by the suprachiasmatic nucleus (SCN) [6]. For instance, Damiola et al. demonstrated that temporal feeding restriction perturbed peripheral circadian oscillators in the liver, kidney, heart, and pancreas without affecting the molecular clock in the SCN [7]. Gómez-Santos et al. also demonstrated the presence of peripheral circadian oscillators in human adipose explants [8].

Among the circadian oscillators, Brain Muscle ARNT-like protein 1 (BMAL1) has been shown to regulate adipocyte differentiation and lipogenesis [6,9]. Indeed, exogenously expressed BMAL1 in 3 T3-L1 adipocytes induced several factors involved with lipogenesis leading to increased lipid synthesis [9]. Conversely, adipose tissue volume was reduced in BMAL1-deficient mice [10].

BMAL1 mRNA expression is known to change dramatically throughout the day, and BMAL1 as well as lipogenic regulatory signals have been shown to be altered by the timing and composition of food intake [5,6,11,12]. Therefore, we hypothesized that daily breakfast skipping might disrupt intracellular molecular clock systems, resulting in changes of the daily cycle of lipogenic protein expression in adipocytes. Furthermore, food intake at the time-points with enhanced lipogenic regulatory signals might increase lipogenesis and lipid storage. The purpose of this study was to examine in 3 T3-L1 adipocytes and assess 1) whether restricted feeding (i.e., “breakfast skipping”) alters the cyclic expression of BMAL1 and lipogenic protein and whether 2) repeated exposure to growth media at the time-points with enhanced lipogenic regulatory signals increases *de novo* lipogenesis and lipid storage.

## Results and discussion

**Figure 1:**
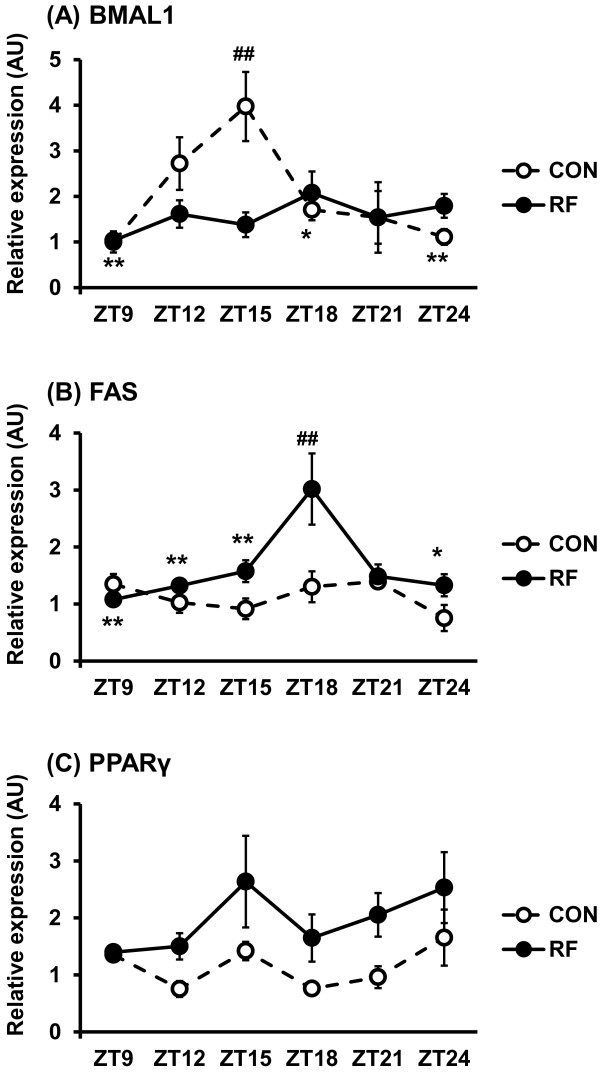
**Cyclic expression of BMAL1 and lipogenic proteins in 3 T3-L1 adipocytes.** Protein expression of BMAL1 (**A**), FAS (**B**), and PPAR-γ (**C**) in the CON group (open circle with a dashed line) and the RF group (closed circle with a solid line) is shown. (**A**) The peak BMAL1 protein expression occurred at ZT 15 in the CON group, but BMAL1 protein expression in the RF group did not change. 2-way ANOVA: time (*p* < 0.05), time by treatment (*p* < 0.01). * *p* < 0.05, ** *p* < 0.01 vs. ZT15 in the CON group. ^##^*p* < 0.01 vs. ZT15 in the RF group. (**B**) The peak FAS protein expression occurred at ZT 18 in the RF group, but FAS protein expression in the CON group did not change. 2-way ANOVA: treatment (*p* < 0.01), time (*p* < 0.01), time by treatment (*p* < 0.05). * *p* < 0.05, ** *p* < 0.01 vs. ZT18 in the RF group. ^##^*p* < 0.01 vs. ZT18 in the CON group. (**C**) PPAR-γ protein expression in the RF group was significantly higher than those in the CON group (*p* < 0.01). 2-way ANOVA: treatment (*p* < 0.01).

We first examined whether restricted feeding (i.e., “breakfast skipping”) could alter the cyclic expression of BMAL1 and lipogenic proteins (e.g., fatty acid synthase (FAS) and peroxisome proliferator-activated receptor gamma (PPAR-γ)) in an *in vitro* model. A peak in BMAL1 protein expression was observed at zeitgeber time (ZT) 15 in the control (CON) group but not in the restricted feeding (RF) group, suggesting that restricted feeding disrupts the intracellular molecular clock system in adipocytes (Figure 1A). In contrast, FAS protein expression did not fluctuate throughout the ZT for the CON group but peaked at ZT 18 for the RF group (Figure 1B). Furthermore, PPAR-γ protein expression in the RF group was significantly higher than that in the CON group (Figure 1C). These results suggest that a bout of restricted feeding (i.e., “breakfast skipping”) could alter the cyclic expression of BMAL1 and increase the expression of lipogenic proteins. One could argue that the *in vitro* model of breakfast skipping in this study is not physiological (e.g. HBSS), thereby inducing autophagy or apoptosis. Thus far, there are no existing *in vitro* models to elucidate the effects of restricted feeding. Although we did not assess autophagy or apoptosis, the 3 T3-L1 adipocytes in this study clearly demonstrated an alteration of their intracellular molecular clock system and a peak of lipogenic protein expression upon restricted feeding. Thus, the 3 T3-L1 adipocytes in this study may be a reasonable *in vitro* model to study the molecular mechanisms underlying augmented lipogenesis during restricted feeding and/or breakfast skipping.

**Figure 2:**
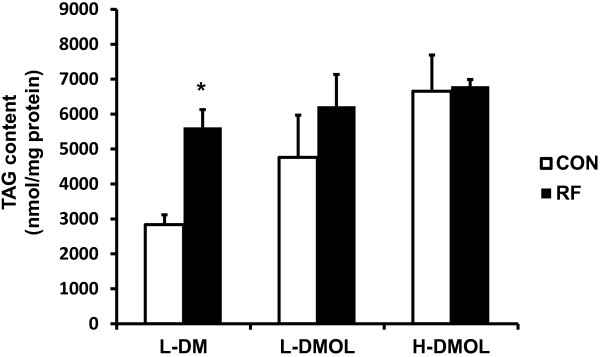
**The effects of repeated exposure to growth media on *de novo *lipogenesis and lipid storage.** Differentiated 3 T3-L1 adipocytes in the CON (open bar) and RF (closed bar) groups (day 9) were incubated from ZT 15 to ZT 19 for 4 consecutive days with either growth medium (L-DM), growth medium with 2 mM oleate (L-DMOL), or DMEM with high glucose/10% FBS supplemented with 5 μg/ml insulin and 2 mM oleate (H-DMOL). TAG content was then measured using a TG E-test kit (Wako). * *p* < 0.05 vs. CON group.

We next examined whether repeated exposure to growth media at the time-points with enhanced lipogenic regulatory signals increases *de novo* lipogenesis and lipid storage. 3 T3-L1 cells were incubated with different media from ZT 15 to ZT 19 for 4 days. Triacylglycerol (TAG) storage in the RF group was significantly higher than that in the CON group when the cells were incubated with the growth medium (L-DM) (Figure 2), suggesting an increased level of lipogenesis. Fatty acid (FA) is synthesized from glucose, and glycerol 3-phosphate, the partner of FA re-esterification and the key substrate for TAG synthesis, is continuously supplied *de novo*, primarily by glycolysis [13]. As the cells were incubated with more calorically rich media, TAG storage in the CON group increased over baseline. As a result, when the cells were incubated with growth medium with 2 mM sodium-oleate (L-DMOL) or DMEM with high glucose/10% FBS supplemented with 5 μg/ml insulin and 2 mM sodium-oleate (H-DMOL), there were no significant differences in TAG storage between the CON and RF groups. Although high glucose supplemented with insulin could promote *de novo* lipogenesis, unsaturated FA, particularly oleate, is normally incorporated into TAG while repressing *de novo* lipogenesis. Therefore, it is plausible that the lipid source in L-DMOL and H-DMOL is the 2 mM sodium-oleate, not solely *de novo* lipogenesis, which might result in no significant difference in the TAG storage between the CON and RF groups. Overall, the results suggest that repeated exposure to growth media at the time-points with enhanced lipogenic regulatory signals increases *de novo* lipogenesis and lipid storage.

In a previous study, Yoshida et al. demonstrated that early nocturnal meal skipping in mice disturbed their peripheral clock and increased *de novo* lipid synthesis in liver and fat tissues. They suggested that increased caloric intake, modulation of peripheral clocks, and alteration in circadian oscillations of core body temperature were involved in the mechanism of lipogenesis [5]. Our experiment strongly indicates that direct disruption of intracellular molecular clock systems as a result of breakfast skipping and concurrent changes in the daily cycle of lipogenic proteins in adipocytes, such as those resulting from repeated exposure to growth media at the time-points with enhanced lipogenic regulatory signals, increases lipogenesis and lipid storage. These alterations are important molecular mechanisms underlying augmented adiposity induced by breakfast skipping. Hence, the timing of food intake could be an attractive target for reducing obesity-associated metabolic disorders. Additionally, physiological stresses to restore disrupted intracellular molecular clock systems warrants further study *in vivo* and *in vitro*.

## Methods

### Cell culture

All cell culture reagents were obtained from Wako (Osaka, Japan) unless otherwise specified. Cell culture protocols were described previously [13]. Briefly, 3 T3-L1 cells were maintained in Dulbecco’s modified Eagle’s medium with low glucose (L-DMEM)/10% fetal bovine serum (FBS). For differentiation, confluent cells (day 0) were treated with a differentiation medium composed of 1 μM dexamethasone, 0.5 mM 3-isobutyl-1-methylxanthine (IBMX), and 5 μg/ml insulin in L-DMEM/10% FBS. After 48 hours (day 2), the differentiation medium was removed, and cells were further cultured in growth medium (L-DMEM/10% FBS supplemented with 5 μg/ml insulin). The growth medium was replaced with the fresh growth medium every 3 days until cell differentiation on day 9 or 11.

### Circadian rhythm induction

From day 11 to 13, differentiated 3 T3-L1 adipocytes were incubated with 3 types of media to synchronize a free-running circadian rhythm. From ZT 9 to ZT 12, cells were incubated with growth medium. From ZT 12 to ZT 15, cells were then incubated with Hank’s Balanced Salt Solution (HBSS)/10% FBS. From ZT 15 to ZT 18, cells were reincubated with growth medium. From ZT 18 to ZT 9 of the next day, cells were incubated with L-DMEM without FBS and insulin.

### Experimental design

On the experimental day (day 13), differentiated 3 T3-L1 adipocytes were divided into two groups, a control group (CON, “breakfast eating”) and a restricted feeding group (RF, “breakfast skipping”). For the CON group, cells were incubated using the aforementioned protocol for circadian rhythm induction. For the RF group, the cell incubation protocol was identical to that for the CON group except growth medium was withheld from ZT 9 to ZT 12. All cells were harvested for Western blotting at ZT 9, 12, 15, 18, 21 and 24.

To examine the second hypothesis, cells in the CON and RF groups were incubated for 4 days (days 9–12) from ZT 15 to ZT 19 with growth medium (L-DM), growth medium with 2 mM sodium-oleate (L-DMOL), or DMEM with high glucose/10% FBS supplemented with 5 μg/ml insulin and 2 mM sodium-oleate (H-DMOL). Cells were harvested on day 13 (ZT 9), and TAG storage was measured. For the RF group, growth medium incubation was withheld from ZT 9 to ZT 12.

### Western blotting

3 T3-L1 cells were washed with PBS and directly dissolved in heated SDS-PAGE sample buffer [13]. Aliquots of the extracts were subjected to SDS-PAGE and transferred to a nitrocellulose membrane. Proteins were probed with antibodies specific for BMAL1 (Millipore, Billerica, MA, USA), FAS (Abcam, Cambridge, MA, USA), PPAR-γ (Abcam), and glyceraldehyde 3-phosphate dehydrogenase (GAPDH) (Sigma-Aldrich, St. Louis, MO, USA) prior to detection via the ECL method (GE Healthcare Life Science, Fairfield, CT, USA).

### Measurement of TAG storage

3 T3-L1 cells were grown in 12-well plates. Differentiated cells were washed twice with Hank’s buffer before harvesting with a lysis buffer (50 mM Tris–HCl, pH 7.4, 150 mM NaCl, 2 mM EDTA, 1% Triton X-100). TAG was measured using a TG E-test kit (Wako) [13].

### Statistical analysis

Differences between two groups were assessed by unpaired Student’s *t*-tests. Multiple parameters were analyzed by two-way analysis of variance (ANOVA) testing. One-way ANOVA and Bonferroni/Dunn post-hoc tests were performed at each time-point when an interaction was observed. *p* values < 0.05 were considered significant. All results are presented as the mean ± S.E.M.

## Abbreviations

ARNT: Aryl hydrocarbon receptor nuclear translocator; BMAL1: Brain and muscle ARNT-like protein 1; SCN: Suprachiasmatic nucleus; ZT: Zeitgeber time; CON: Control; RF: Restricted feeding; FAS: Fatty acid synthase; PPAR-γ: Peroxisome proliferator-activated receptor gamma; DMEM: Dulbecco’s modified Eagle medium; FBS: Fetal bovine serum; IBMX: 3-isobutyl-1-methylxanthine; L-DM: Growth medium with low glucose; L-DMOL: Growth medium with 2 mM sodium-oleate; H-DMOL: DMEM with high glucose/10% FBS supplemented with 5 μg/ml insulin and 2 mM sodium-oleate; HBSS: Hank’s Balanced Salt Solution.

## Competing interest

Both authors declare no conflicts of interest.

## Authors’ contributions

TH and YE designed and performed the experiments. Both authors read and approved the final manuscript.
